# Supplementary material for: Causal Relationship between Aging and Anorexia Nervosa: A White-Matter-Microstructure-Mediated Mendelian Randomization Analysis
Source: Biomedicines. 2024 Aug 16;12(8):1874. doi: 10.3390/biomedicines12081874 (PMC11351342; doi:10.3390/biomedicines12081874)
Supplement: Supplementary file 1 [file biomedicines-12-01874-s001.zip › Supplementary Figure/Figure S4/Singe SNP plot of MR analysis for TL to ubm-b-1615.pdf]

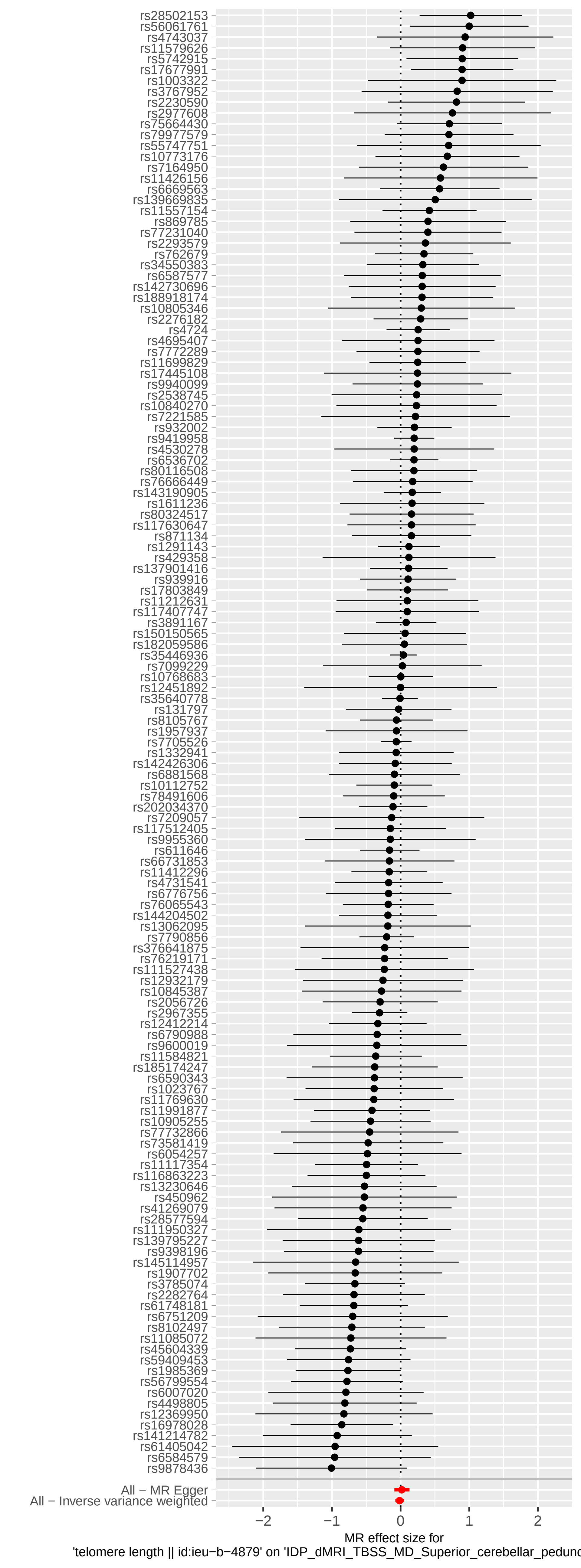
rs28502153
rs56061761
rs4743037
rs11579626
rs5742915
rs17677991
rs1003322
rs3767952
rs2230590
rs2977608
rs75664430
rs79977579
rs55747751
rs10773176
rs7164950
rs11426156
rs6669563
rs139669835
rs11557154
rs869785
rs77231040
rs2293579
rs762679
rs34550383
rs6587577
rs142730696
rs188918174
rs10805346
rs2276182
rs4724
rs4695407
rs7772289
rs11699829
rs17445108
rs9940099
rs2538745
rs10840270
rs7221585
rs932002
rs9419958
rs4530278
rs6536702
rs80116508
rs76666449
rs143190905
rs1611236
rs80324517
rs117630647
rs871134
rs1291143
rs429358
rs137901416
rs939916
rs17803849
rs11212631
rs117407747
rs3891167
rs150150565
rs182059586
rs35446936
rs7099229
rs10768683
rs12451892
rs35640778
rs131797
rs8105767
rs1957937
rs7705526
rs1332941
rs142426306
rs6881568
rs10112752
rs78491606
rs202034370
rs7209057
rs117512405
rs9955360
rs611646
rs66731853
rs11412296
rs4731541
rs6776756
rs76065543
rs144204502
rs13062095
rs7790856
rs376641875
rs76219171
rs111527438
rs12932179
rs10845387
rs2056726
rs2967355
rs12412214
rs6790988
rs9600019
rs11584821
rs185174247
rs6590343
rs1023767
rs11769630
rs11991877
rs10905255
rs77732866
rs73581419
rs6054257
rs11117354
rs116863223
rs13230646
rs450962
rs41269079
rs28577594
rs111950327
rs139795227
rs9398196
rs145114957
rs1907702
rs3785074
rs2282764
rs61748181
rs6751209
rs8102497
rs11085072
rs45604339
rs59409453
rs1985369
rs56799554
rs6007020
rs4498805
rs12369950
rs16978028
rs141214782
rs61405042
rs6584579
rs9878436
All − MR Egger
All − Inverse variance weighted
−2
−1
0
1
2
MR effect size for
'telomere length || id:ieu−b−4879' on 'IDP_dMRI_TBSS_MD_Superior_cerebellar_pedunc
